# Costs of care for persons with opioid dependence in commercial integrated health systems

**DOI:** 10.1186/1940-0640-9-16

**Published:** 2014-08-14

**Authors:** Frances L Lynch, Dennis McCarty, Jennifer Mertens, Nancy A Perrin, Carla A Green, Sujaya Parthasarathy, John F Dickerson, Bradley M Anderson, David Pating

**Affiliations:** 1 Kaiser Permanente Center for Health Research, 3800 N. Interstate Avenue, Portland, OR 97227, USA; 2 Department of Public Health & Preventive Medicine, Oregon Health & Science University, 3181 S.W. Sam Jackson Hill Road, CB 669, Portland, OR 97239, USA; 3 Kaiser Permanente Division of Research, 2000 Broadway, Oakland, CA 94612, USA; 4 Addiction Medicine Department, Kaiser Permanente Northwest, 3550N. Interstate Avenue, Portland, OR 97227, USA; 5 Kaiser Permanente Chemical Dependency Recovery Program, 1201 Fillmore Street, San Francisco, CA 94115, USA

**Keywords:** Substance abuse, Cost analysis, Health care utilization, Commercial health insurance, Parity

## Abstract

**Background:**

When used in general medical practices, buprenorphine is an effective treatment for opioid dependence, yet little is known about how use of buprenorphine affects the utilization and cost of health care in commercial health systems.

**Methods:**

The objective of this retrospective cohort study was to examine how buprenorphine affects patterns of medical care, addiction medicine services, and costs from the health system perspective. Individuals with two or more opioid-dependence diagnoses per year, in two large health systems (System A: n = 1836; System B: n = 4204) over the time span 2007–2008 were included. Propensity scores were used to help adjust for group differences.

**Results:**

Patients receiving buprenorphine plus addiction counseling had significantly lower total health care costs than patients with little or no addiction treatment (mean health care costs with buprenorphine treatment = $13,578; vs. mean health care costs with no addiction treatment = $31,055; p < .0001), while those receiving buprenorphine plus addiction counseling and those with addiction counseling only did not differ significantly in total health care costs (mean costs with counseling only: $17,017; p = .5897). In comparison to patients receiving buprenorphine plus counseling, those with little or no addiction treatment had significantly greater use of primary care (p < .001), other medical visits (p = .001), and emergency services (p = .020). Patients with counseling only (compared to patients with buprenorphine plus counseling) used less inpatient detoxification (p < .001), and had significantly more PC visits (p = .001), other medical visits (p = .005), and mental health visits (p = .002).

**Conclusions:**

Buprenorphine is a viable alternative to other treatment approaches for opioid dependence in commercial integrated health systems, with total costs of health care similar to abstinence-based counseling. Patients with buprenorphine plus counseling had reduced use of general medical services compared to the alternatives.

## Background

Opioid abuse and dependence, whether through use of heroin or prescription opioid medications, has high costs to individuals, health care systems, and society. Total costs, including health care, lost productivity, and costs of crime, are more than $28 billion per year, with health care alone costing over $8 billion [1,2]. Costs are increasing, moreover, because of the increased prevalence of opioid dependence [3,4] and its associated disease burdens (e.g., increased risk of HIV, hepatitis C), as well as its emotional and financial impacts on families [5,6].

Federal legislation permits new options in agonist therapy. Specifically, the Drug Addiction Treatment Act of 2000 allows qualified physicians (those with 8 hours of opioid-dependence treatment training) to request a waiver to prescribe opioid-agonist medications with Food and Drug Administration (FDA) approval. In 2002, the FDA approved two forms of buprenorphine (a partial opioid agonist)—Subutex ® (buprenorphine alone) and Suboxone ® (buprenorphine and naloxone). The buprenorphine/naloxone (buprenorphine) combination is designed to reduce abuse potential and is the medication typically used in primary care settings. Cochrane Reviews document the effectiveness of methadone maintenance [7] and buprenorphine [8] for long-term pharmacotherapy to treat opioid dependence. A review of the literature indicates that while methadone maintenance may be slightly more effective than buprenorphine maintenance, buprenorphine may reduce mortality and has the added advantage that the therapy can be managed in primary care settings [3].

Expansion of access to buprenorphine in general medical systems could extend agonist therapy to many untreated opioid-dependent persons who either do not have access to a methadone clinic or who do not want the burden of daily medication visits. In addition, expanding access may improve the quality of care for opioid-dependent individuals, because physicians in general medical settings may identify and manage co-occurring health consequences of opioid dependence as part of monitoring the agonist pharmacotherapy [9,10].

Most commercial health systems, however, have little experience with providing opioid-agonist therapy [11]. Information on the cost of alternative treatments is particularly salient, as health systems face increased competition, pressure to reduce costs, and increasing requirements to provide comprehensive mental health and addiction treatments [12,13]. Health systems need to understand the direct cost of the intervention, as well as its impact on other health care costs. While the direct cost of buprenorphine is significantly higher than the cost of methadone [14,15], it is less clear how buprenorphine affects other health care spending. Office-based buprenorphine treatment, for example, may decrease the need for emergency department visits, increase appropriate contact with primary care and, as a result, increase identification of and treatment for comorbid conditions [16], which might be more costly initially, but might also improve health outcomes and reduce long-term costs.

Research on patterns of the cost and cost-effectiveness of buprenorphine compared to alternative therapies has been mixed. Studies in publicly funded health systems (Veterans Administration, Medicaid) reported lower costs for buprenorphine patients compared to methadone patients [14,17]; commercial health systems, however, might experience different costs. Buprenorphine appears to be cost-effective under certain scenarios [18,19], yet other studies suggest that methadone maintenance is more cost-effective [15,20,21]. None of these studies examined the cost or cost-effectiveness of buprenorphine in a commercially insured population. In addition, prior studies have not assessed the incremental cost of buprenorphine in combination with counseling, compared to counseling only (without opioid-replacement medication), which is the primary form of care for substance abuse in many private commercial health systems.

The focus of this paper is to provide information for health system decisionmakers about the impact of buprenorphine treatment for opioid dependence in commercial health systems. We assessed the utilization and health care costs for opioid-dependent patients and compared patients receiving buprenorphine plus counseling from addiction medicine providers to opioid-dependent patients receiving counseling only, and to opioid-dependent patients receiving little or no addiction treatment. We address two research questions: 1) Are total health system costs different for persons treated with buprenorphine plus counseling, compared with those who are treated with counseling only and those receiving little or no addiction treatment? 2) Are patterns of addiction treatment and other medical care services different for persons treated with buprenorphine plus counseling, compared with those who are treated with counseling only or those with little or no addiction treatment?

## Methods

We conducted a retrospective cohort study using data from two large, nonprofit, integrated health systems that provide addiction medicine treatment and comprehensive medical care. Patients were categorized into one of three treatment groups: buprenorphine plus counseling, counseling only (two or more counseling sessions and no medication), and little or no addiction treatment (one or fewer counseling sessions and no medication). We used multivariate analyses to assess health care utilization patterns and total costs for 2007–2008. Both health systems had implemented buprenorphine treatment and had stable patient populations during this time period. Propensity scores helped control for differences in patient characteristics that could be related to type of treatment received.

### Addiction medicine services: health system A

In Health System A, persons presenting with opioid-dependence diagnoses receive a medical assessment to determine the level of care needed. Depending on client preference and the medical provider’s assessment, the patient may or may not receive agonist medication. All patients, whether treated with agonist therapy or not, are strongly encouraged to attend addiction counseling. Patients who refuse addiction counseling may be discontinued from agonist medication. Addiction medicine services include residential treatment, specialty outpatient groups for persons with opioid dependence, general outpatient counseling groups, and individual therapy. Patients receiving buprenorphine participate in case management and sign a treatment agreement that states the expectations for continued buprenorphine care. Frequent, random, urine drug screens are required.

### Addiction medicine services: health system B

In Health System B, patients with chemical dependency, including opioid dependence, receive an individualized clinical assessment. Outpatient, day-hospital, or residential treatment is provided. Day treatment is more intensive, generally including four times the amount of each service as outpatient treatment, although the content is the same. Patients using buprenorphine are integrated with other patients in the group-based program and also attend buprenorphine-specific groups. Patients are expected to attend 12-step meetings off-site. Patients receive frequent, random breathalyzer and urine drug screens throughout their treatment.

## Study sample

We identified individuals with two or more health system encounters with diagnoses of opioid dependence, using clinical and administrative data for the calendar years 2007–2008. All identified members had at least one month of health system eligibility in the year prior to the study. We included all service use and related cost data for services paid for by the health systems, including care provided directly through the health systems and claims for services provided outside of the health systems. We examined the use and cost of addiction treatment (including inpatient treatment, residential treatment, and outpatient counseling) and other medical services (primary care, emergency services, mental health services, and other non-addiction medical visits) during the study period. Both inpatient and outpatient costs outside the health system were obtained from outside claims and referral systems and were based on the amount the health system actually paid to the vendor, versus the amount billed to the vendor. Prescription costs were obtained from internal data from the health systems and approximate retail costs (acquisition cost, plus dispensing fee). We used the Consumer Price Index to adjust for inflation.

To control for possible differences in health conditions related to type of treatment received, we categorized all diagnoses for each member in the year prior to the opioid diagnosis using a disease classification system (CBC) [22], updated for ICD-9-CM codes. This system organizes health problems into groups that share important dimensions, including severity, etiology, duration, and anticipated use of medical resources. The system has 19 primary categories (e.g., chronic disease, serious; microorganism, less serious). All disease codes and V codes are assigned to a single appropriate category, simplifying diagnosis-related analyses, and then coded 0 for none in the category or 1 for at least one event in the category.

Institutional Review Boards (IRBs) for the two health systems reviewed and approved the study protocol. Oregon Health & Science University’s review board ceded oversight of the study to the two health systems’ IRBs, because the study analyzed health system records.

## Analysis

Multivariate analyses examined patterns of health service use and cost by comparing the different treatment modality groups. Because treatment assignment was observed (and not randomized), we created propensity scores using multinomial regression, predicting type of treatment received (with buprenorphine as the reference group) to help control for systematic differences in the treatment modality groups. The propensity score model included the 19 CBC scores in the prior year, number of opioid diagnoses in the prior year, six variables for health care utilization in the prior year (detoxification stays, inpatient stays, primary care visits, emergency room visits, mental health visits, and other/specialty visits), number of days in the prior year on buprenorphine, the Charlson comorbidity score for the year prior, months of health system eligibility in the year prior, age, gender, and site. Since we used multinomial regression with three levels of the outcome variable, each person received three propensity scores. To examine the fit of the model, each person was classified into the group associated with the highest propensity score and computed percent correct classification. To examine the balance of propensity scores, we computed the median standardized difference across the 42 covariates [23]. We were interested in the impact on costs and utilization from a health plan perspective; therefore, we did not use propensity score matching, as that would have limited the generalizability to only those cases that could be matched. Because each person received three propensity scores, we did not use inverse probability weighting and instead, included the propensity scores as covariates in the main analyses (the propensity scores sum to 1.0, so we included two of the three propensity scores). The same individuals could appear in more than one year; therefore, we used generalized estimating equations (GEE) to model the covariance structure and test for differences in utilization among the study groups, controlling for propensity of type of treatment received, site, age, gender, and months of eligibility in the health system. We used Poisson and negative binomial models with a log-link function to examine health care utilization variables. Our analyses of inpatient detoxification used only those patients who were not on Medicaid (n = 5757), because inpatient detoxification services were not part of the Medicaid-contracted services with the health systems. All other utilization analyses included all patients (N = 6040) and Medicaid status as an additional covariate. A generalized linear model with gamma distribution, log-link function, and robust standard errors was used to analyze total costs. Cost analyses were adjusted to 2008 dollars. Total costs included addiction treatment services, emergency, inpatient, primary care, all other specialty care medical services (e.g., mental health care), and pharmaceuticals.

## Results

Table 1 summarizes characteristics of the population samples from the two health plans; 52 percent of the patients were female, and the mean age was 42 years. Overall, the three patient groups were relatively similar in total numbers and percentage: 30 percent with little or no addiction treatment, 32 percent with buprenorphine plus counseling, and 38 percent with counseling only. The two health plans, however, differed in the use of buprenorphine; in Health Plan A, 45 percent of the opioid-dependent patients received buprenorphine, versus 27 percent in Health Plan B.

**Table 1:** Sample characteristics and sample sizes for each health system

	**Health system A**	**Health system B**	**Total**
	**n = 1836**	**n = 4204**	**N = 6040**
Age in years, Mean (SD)	39 (15)	43 (14)	42 (14)
Gender,% female	50%	52%	52%
Counseling only, n	499	1781	2280
Little or no addiction treatment, n	513	1301	1814
Buprenorphine plus counseling, n	824	1122	1946

### Propensity model

The model correctly predicted group membership for 60.3 percent of the cases, where 33.3 percent correct classification would be expected by chance alone. Correct classification was similar for the three groups (counseling only – 61.0%, little or no treatment – 62.4%, buprenorphine plus counseling – 57.7%). The standardized median difference (a measure of balance of the predictors across the groups) for the two propensity scores were .18 and .35. Values < .25 were considered an acceptable fit [23]. This is particularly important when the propensity scores are used for matching or stratification. However, the propensity scores were not used for matching or stratification in this study, but were included as covariates in the main analysis, which helped to mitigate any lack of balance in the second propensity score. Age, gender, comorbidity, and prior health care were the strongest predictors in the model. Table 2 summarizes the three groups on these variables. In general, the buprenorphine group was younger, less likely to be female, had fewer comorbidities, had more previous diagnoses of opioid dependency, had more addiction medicine contacts, and more previous days on buprenorphine.

**Table 2:** Mean (SD) characteristics and utilization in the year prior to study by treatment group

	**Counseling only**	**Little or no addiction treatment**	**Buprenorphine plus counseling**
Age	40.95 (13.10)	48.65 (15.14)	36.02 (12.46)
Female (%)	51%	62%	43%
Number of opioid dx	5.00 (9.54)	2.94 (6.59)	9.85 (12.52)
Number of primary care visits	8.27 (17.19)	13.19 (28.40)	5.10 (6.53)
Number of outpatient addiction medicine visits	16.15 (36.67)	1.96 (11.60)	18.31 (40.52)
Days on buprenorphine	2.81 (21.87)	1.55 (16.65)	72.70 (126.86)
Charlson comorbidity score	0.32 (0.59)	0.67 (0.99)	0.18 (0.46)

### Costs

Table 3 presents the adjusted mean total health care costs per person, per year, by group. Patients receiving buprenorphine plus counseling had significantly lower total health care costs ($13,578; 95% CI: $13,364–$13,791) than those with little or no treatment ($31,035; 95% CI: $30,433–$31,637; p < .001). Total health care costs did not differ significantly between those receiving buprenorphine plus counseling and those with counseling only ($17,017; 95% CI: $16,751–$17,285); p = .569).

**Table 3:** Adjusted mean annual cost 2008 U.S. dollars

	**Mean annual cost (95% CI)**
Counseling only	17,017^t^
	(16,751 – 17,285)
Little or no addiction treatment	31,035^**^
	(30,433 – 31,637)
Buprenorphine plus counseling	13,578
	(13,364 – 13,791)

Costs are adjusted for age, gender, months of eligibility in the health system, site, and propensity of type of treatment received.

t – Not significantly different from buprenorphine plus counseling at the .05 level.

**Significantly different from buprenorphine plus counseling at p < .001.

### Patterns of service use

Table 4 presents the patterns of health care utilization by treatment group. In comparison to the buprenorphine plus counseling group, the group with little or no addiction treatment had significantly greater use of primary care (z = 4.33, p < .001), other medical visits (z = 3.24, p = .001), and emergency services (z = 2.33, p = .020). Patients with counseling only (compared to patients with buprenorphine plus counseling) used less inpatient detoxification (z = -8.57, p <. 001), and had significantly more PC visits (z = 3.41, p = .001), other medical visits (z = 2.84, p = .005), and mental health visits (z = 3.09, p = .002). The counseling only and buprenorphine plus counseling groups did not differ significantly on use of residential treatment or emergency services.

**Table 4:** Mean health care utilization (SE) by treatment group

	**Inpatient detox**	**Residential stays**	**PC visits**	**ER visits**	**MH visits**	**Other visits**
Counseling only	.05^*^	.07^t^	7.92^*^	1.25^t^	3.30^*^	5.81^*^
	(.12)	(.10)	(.06)	(.06)	(.09)	(.04)
Little or no addiction treatment	.004*	.01*	7.87*	1.45^*^	2.94^t^	6.22^*^
	(.31)	(.36)	(.04)	(.06)	(.12)	(.05)
Buprenorphine plus counseling	.07	.07	6.15	1.10	2.33	4.97
	(.11)	(.11)	(.03)	(.08)	(.09)	(.04)

Estimates are adjusted for age, gender, months of eligibility in the health system, Medicaid status, site, and propensity of type of treatment received.

t – Not significantly different from buprenorphine plus counseling at the .05 level.

*Significantly different from buprenorphine plus counseling at p < .05.

## Discussion

Access to buprenorphine treatment for opioid dependence has increased and is likely to continue to increase because the Mental Health Parity and Addiction Equity Act and the Affordable Care Act enhance health insurance coverage for mental health and addiction treatment services. Commercial health systems caring for persons with opioid dependence need to develop treatment services to promote the best possible outcomes within highly constrained budgets. Results from this study suggest that health system costs for persons treated with buprenorphine plus counseling were similar to health system costs for persons treated with counseling only, and about $17,000 per year less than the health system costs for patients with little or no addiction treatment. The availability of generic buprenorphine/naloxone, moreover, should reduce costs for the buprenorphine plus counseling group in the future. Patients with little or no addiction treatment had significantly greater use of medical services; notably, emergency services. Opioid-dependent patients not engaged in addiction treatment may continue to have significant health consequences related to opioid use. In contrast, the patients with buprenorphine plus counseling and counseling only had similar lower levels of emergency services, suggesting that their addiction treatment reduced the need for crisis care. Patients with counseling only used more primary care services, mental health services, and other medical visits than patients with buprenorphine plus counseling. Reductions in the use of primary and other medical care services amongst buprenorphine plus counseling patients may reflect additional care coordination or advice received during medication management visits.

The two health systems participating in this study both provide comprehensive health care services to commercially insured populations, much of which are directly provided by the health systems. At the time of the study, both systems had set up infrastructure to support buprenorphine services (physician and counselor training, and pharmacy access) and had increased the number and percentage of patients receiving buprenorphine [24]. However, they were both relatively new to managing buprenorphine treatment. System B, in particular, had a more complex system of providers across a larger geographic area. Uptake and implementation was slower in System B, and System B had less prior experience with opioid-agonist therapy. The health system costs associated with buprenorphine treatment may change over time, as health systems become more experienced and efficient at providing opioid-agonist therapy.

Buprenorphine treatment provides clinical outcomes that are similar or better than other addiction medicine treatment approaches for persons with opioid dependence. Most prior studies, however, focused on the use of buprenorphine in publicly insured populations. Results of this study provide evidence that buprenorphine treatment can be provided at a cost similar to abstinence-based counseling in commercial health systems. In addition, buprenorphine may have three advantages compared to alternative treatments. Convenience is a major consideration; patients treated with buprenorphine are not required to go to a specialized clinic every day to receive medication, and general medical offices tend to be closer to patient’s homes, with lower patient travel costs. Second, patients often prefer the medical office treatment environment because of their familiarity with providers and facilities and their trust in providers to help them address medical conditions related to opioid use. Finally, patients report that medical settings provide a better environment to maintain sobriety and avoid others who are current users of illicit drugs [24].

### Limitations

Interpretation of our results should be considered in light of several limitations. The current study used data from two large integrated health systems that use comprehensive and detailed electronic medical records. Although the diagnostic data is very likely to be accurate, we were not able to group people by how long they had been in treatment for opioid dependence, which could influence health care costs. Our retrospective cohort analysis did not randomize participants to treatment modality. We addressed this issue by using propensity scores obtained from a model that included prior health care utilization and other important factors to adjust for differences between groups related to selection into specific treatment modalities. Although this approach could help to control for observable differences, it did not address differences from unobservable factors and therefore, causal inferences cannot be drawn. The measure of balance of the predictors across groups met the preferred level (< .25 for goodness of fit [23]) for one of the propensity scores, but was .35 for the second propensity score. However, this measure is most important for matching and stratification and in this study, the propensity scores were used as covariates helping to mitigate any lack of balance in this score. An additional concern with use of propensity scores for covariate adjustment is that it requires an assumption that outcomes of the regression are correctly specified. These limitations may lead to a bias in the magnitude of effects detected, although this issue is less of a concern in the context of larger samples (e.g., sample size in the 1000s) [25,26]. Both integrated health systems are located in the Western United States, so results may not be generalizable to other populations or geographic regions. On the other hand, the analysis included all patients with opioid-dependence diagnoses enrolled in two large integrated health systems, so our results are likely to represent what these types of private, integrated, health systems experience in practice. This study focused on providing information that could be useful to health system decisionmakers. However, a health system perspective does not include some important nonhealth social costs. For example, criminal justice costs might be reduced by the increased use of buprenorphine treatment.

## Conclusions

Results of this study suggest that buprenorphine can be offered in integrated health systems to commercially insured patients at total health care costs that are similar to those seen with abstinence-based counseling programs. Patients treated with buprenorphine plus counseling had reduced use of general medical services compared to those treated with alternative approaches. Future research needs to examine the long-term costs related to alternative treatments for opioid dependence, both from the health system and the patient perspective.

## Competing interests

The authors declare they have no competing interests.

## Authors’ contributions

FLL contributed to the design of the study, collection of data, design of statistical analyses, interpretation of the data, and led the writing of the manuscript. DM contributed to the design of the study, collection of data, interpretation of data, and helped to draft the manuscript. JM contributed to the design of the study, collection of data, interpretation of the data, and helped to draft the manuscript. NAP contributed to the design of the study, collection of data, design of the statistical analyses, conduct of the statistical analyses, interpretation of the data, and helped to draft the manuscript. CAG contributed to the design of the study, collection of data, and interpretation of the data. SP contributed to the design of the study, collection of data, and interpretation of the data. JFD contributed to the design of the statistical analyses, conduct of statistical analyses, and interpretation of the data. BMA contributed to the design of the study, collection of data, and interpretation of the data. DP contributed to the collection of data and interpretation of the data. All authors read and approved the final manuscript.
